# Association of C1QTNF6 gene polymorphism with risk and clinical features of type 1 diabetes in Chinese: implications for ZnT8A and beta-cell function

**DOI:** 10.3389/fimmu.2025.1551552

**Published:** 2025-04-09

**Authors:** Jinhan Liu, Ying Xia, Zhiguo Xie, Xia Li, Gan Huang, Jingyi Hu, Zhiguang Zhou

**Affiliations:** National Clinical Research Center for Metabolic Diseases, Key Laboratory of Diabetes Immunology (Central South University), Ministry of Education, and Department of Metabolism and Endocrinology, The Second Xiangya Hospital of Central South University, Changsha, Hunan, China

**Keywords:** type 1 diabetes, Genetics, C1QTNF6, Autoimmunity, Beta-cell function

## Abstract

**Introduction:**

Genome-wide association study identified *C1QTNF6* as a candidate gene for type 1 diabetes (T1D) in Caucasians. We aimed to investigate if rs229541 in *C1QTNF6* conferred susceptibility to T1D in Chinese, independent of *DR-DQ* genotypes and if this gene polymorphism affected the clinical profiles of T1D.

**Methods:**

In this case–control study, genotypes of *C1QTNF6* rs229541 were obtained from 1278 patients with T1D and 1282 nondiabetic controls using MassARRAY.

**Results:**

Genotypic (P = 0.0210) and allelic (P = 0.0084) frequencies were significantly different between the T1D group and the control group. When the model was adjusted for *DR-DQ* genotypes, G allele carriers were observed less often in the T1D group (P = 0.0423, OR 0.82, 95% CI 0.68-0.99) than in the control group, and the G allele was associated with reduced T1D risk(P = 0.0167, OR 0.83, 95% CI 0.71-0.97). T1D patients who were homozygous for the G allele showed a higher positive rate of ZnT8A than carriers of the A allele (P = 0.0171, OR 1.88, 95% CI 1.12-3.16). By detection of fasting C-peptide, G allele carriers exhibited a lower frequency of beta-cell failure compared to those with A/A genotype (P = 0.0058, OR 0.70, 95% CI 0.54-0.90). *C1QTNF6* was not found to be correlated with GADA, IA-2A or age at T1D diagnosis.

**Discussion:**

The polymorphism in *C1QTNF6* was independently associated with T1D risk in Chinese and broadly modified clinical features of the disease. This loci might be utilized to construct genetic risk model in combination with the well-known *DR-DQ* region for future screening of genetically T1D prone individuals among Chinese.

## 1 Introduction

Type 1 diabetes (T1D) is generally considered to be an endocrine disorder initiated by an autoimmune attack targeting pancreatic beta-cells, and susceptibility to T1D can be attributed to both genetic and environmental elements (1). In addition to the predominant genetic determinants of T1D risk located within the HLA region (1), over 70 non-HLA loci that confer modest susceptibility to T1D individually have been identified in several genome-wide association studies and meta-analyses over the past 14 years (2–4). Furthermore, these findings have already been used into clinical practice: a genetic risk score constructed by a cluster of non-HLA SNPs in combination with *DR-DQ* information has shown efficacy in discriminating between diabetes subtypes and new-born screening for T1D-susceptible individuals (5).

However, the abovementioned associations with T1D are mostly reported in Caucasians and cannot be directly applied to other ethnic groups. For instance, the mutant T allele of the well-established *PTPN22* has rarely been observed in Asian populations (6), whereas the *DR9* haplotype is popular in East Asian patients and rare among Caucasians (7). Hence, the notable heterogeneity of T1D across diverse populations indicates the need to delineate the genetics of this disease in non-Caucasian populations, as association studies of gene polymorphisms are rare in these groups. For Chinese patients, only a limited number of genes have been identified as candidates for T1D, including *PTPN22* (6), *CTLA4* (8), *ERBB3* (9) and *STAT4* (10). Thus, the vast majority of non-HLA genes that indicate T1D risk among Caucasians remain unverified in Chinese Han individuals.

Gene polymorphism rs229541, an intron of complement 1q tumour necrosis factor-related protein 6 (*C1QTNF6*, also known as *CTRP6*), was identified as a T1D-related locus in a prior genome-wide association study among Caucasians (2). The protein encoded by *C1QTNF6* has proven to be a pattern recognition molecule that binds to a wide variety of endogenous or microorganic ligands, followed by recruitment of collectin-11. It subsequently initiates the complement cascade and decomposition of C4 and is thus involved in the immune response (11). Overlapping risk variants within *C1QTNF6* have been found among various autoimmune disorders, evidenced by rs229541 in rheumatoid arthritis (12), rs229527 (in tight linkage disequilibrium with rs229541) in generalized vitiligo (13) and rs229527 in Graves’ disease (14), indicating a common molecular pathway underlying these diseases. To the best of our knowledge, the role of *C1QTNF6* polymorphisms in the etiopathogenesis of T1D among non-Caucasian populations has not been validated thus far.

Therefore, the current research was carried out to determine 1. whether *C1QTNF6* rs229541 conferred susceptibility to T1D of Chinese origin; 2. whether the effect stemmed from the polymorphism itself or was merely a reflection of the uneven distribution of *DR-DQ* genotypes among participants; and 3. whether this variant modulated clinical profiles of T1D, including autoantibody status, age at diagnosis and beta-cell function.

## 2 Materials and methods

### 2.1 Study participants

A total of 1280 T1D patients were identified in the Department of Metabolism and Endocrinology of the Second Xiangya Hospital. The inclusion criteria were as follows: 1. diagnosis of diabetes based on the criteria put forth by the WHO in 1999; 2. acute disease onset and dependency on insulin administration after diagnosis; and 3. positivity of at least one of the three islet autoantibodies, i.e., glutamic acid decarboxylase antibody (GADA), protein tyrosine phosphatase antibody (IA-2A), and zinc transporter 8 antibody (ZnT8A). A total of 1293 control subjects with normal glucose tolerance but without a family history of diabetes were recruited from the Department of Physical Examination in the Second Xiangya Hospital. All the participants declared themselves to be of Chinese Han ethnicity. This research was approved by the Ethics Committee of the Second Xiangya Hospital and was conducted in line with the Declaration of Helsinki guidelines. All the participants or guardians of children with T1D gave their written informed consent and were completely aware of the study procedures.

### 2.2 Biochemical assays and genotyping approach

Both fasting and postprandial C-peptide levels were detected with a chemiluminescence approach from the Adiva Centaur System kit. Approximately 70% of T1D subjects were tested within 1 year of disease onset, while the remaining 30% were tested at least 1 year after disease onset. Radioligand assays were performed in duplicate to detect autoantibodies against islets, including GADA, IA-2A and ZnT8A. All T1D patients underwent these assays at diagnosis, and positivity was confirmed by repetition to ensure that case subjects negative for all autoantibodies and those with equivocal results were not enrolled. MassARRAY (Sequenom, the US) was applied to genotype *C1QTNF6* rs229541 followed by quality control, in which 2 samples from T1D patients and 11 samples from control subjects failed and were excluded from statistical analysis, to ensure the accuracy and reliability of the data analysis. The overall detection rate for this SNP was 99.49%. The methods of HLA genotyping were described in a previous publication by our group (15), and *DRB1-DQA1-DQB1* haplotypes were constructed using PHASE version 2.0 (16). HLA haplotypes other than the susceptible DR3, DR4 and DR9 were integrated and defined as DRX.

### 2.3 Statistical analysis

Data were all computed in SPSS version 22.0 (IBM, USA). Continuous variables with normal distributions were expressed as the mean ± standard deviation and compared via Student’s t test, whereas nonnormally distributed variables were expressed as the median and interquartile range and compared via the Mann–Whitney U test. Categorical variables were expressed as n (%) and compared using the chi-squared test. The chi-squared test was also performed to directly compare genotypic and allelic frequencies with the Hardy-Weinberg equilibrium. The following analyses were all conducted using stepwise logistic regression, which included DRB1-DQA1-DQB1 genotypes (DRX/DRX as the reference) as covariates to adjust for HLA information. Under the assumption of a dominant model, G/A and G/G genotypes were encoded as 1 and A/A genotype as 0. In the recessive model, G/A and A/A genotypes were set as 0 and G/G as 1. For the additive model, which calculated the effect for each copy of the minor allele G, the A/A genotype was defined as 0, G/A as 1 and G/G as 2. When investigating the clinical relevance of gene polymorphism, positivity of islet autoantibodies, early disease onset and worse beta-cell function were encoded as 1, and negativity of islet autoantibodies, late disease onset and better beta-cell function were encoded as 0. P <0.05 was considered statistically significant.

## 3 Results

### 3.1 Sample description

Demographic and clinical characteristics for 1278 T1D subjects and 1282 nondiabetic controls included in the further analysis are summarized in **Table 1**. Briefly, there were no significant differences in sex between the two groups (*P =* 0.9196), and T1D subjects were younger (*P <* 0.0001). As expected, T1D patients were remarkably leaner (*P <* 0.0001) and had higher levels of fasting plasma glucose (*P <* 0.0001) and 2-hour postprandial plasma glucose (*P <* 0.0001) than controls. The median age at onset among the T1D group was 21.0 (12.0-34.0) years, and 86.5% of the patients manifested diabetic ketoacidosis when the disease occurred. The patients showed an average HbA1c level of 10.5 ± 3.7%, a median fasting C-peptide level of 82.6 (29.5-167.0) pmol/L and a median postprandial C-peptide level of 163.6 (54.9-340.5) pmol/L. The disease duration was documented when C-peptide levels were determined. GADA, IA-2A and ZnT8A were positive in 90.2%, 49.5% and 34.5% of the T1D patients, respectively.

**Table 1:** Clinical characteristics of patients with T1D and control subjects.

	Control subjects n = 1282	T1D patients n = 1278	*P*
Male (n, %)	686 (53.8)	671 (53.6)	0.9161
Age (years)	42.0 (29.0-51.0)	24.0 (13.0-36.0)	<0.0001
BMI (kg/m^2^)	23.0 ± 3.4	19.1 ± 3.6	<0.0001
FPG (mmol/L)	4.9 ± 0.6	10.2 ± 5.6	<0.0001
PPG (mmol/L)	5.6 ± 1.2	16.2 ± 7.4	<0.0001
Age at onset (years)		21.0 (12.0-34.0)	
Diabetic ketoacidosis		1008 (86.5)	
HbA1c (%)		10.5 ± 3.7	
Disease duration (months)		3.0 (0.5-15.3)	
FCP (pmol/L)		82.6 (29.5-167.0)	
PCP (pmol/L)		163.6 (54.9-340.5)	
GADA positivity (n, %)		1151 (90.2)	
IA-2A positivity (n, %)		494 (49.5)	
ZnT8A positivity (n, %)		298 (34.5)	

Sex and positivity of islet autoantibodies are expressed as n (%). Age at onset, FCP and PCP are expressed as medians with interquartile ranges, while the other variables are expressed as the mean ± standard deviation. T1D, type 1 diabetes; BMI, body mass index; FPG, fasting plasma glucose; PPG, 2-hour postprandial plasma glucose; HbA1c, glycosylated hemoglobin; FCP, fasting C-peptide; PCP, 2-hour postprandial C-peptide; GADA, autoantibody against glutamic acid decarboxylase; IA-2A, autoantibody against protein tyrosine phosphatase; ZnT8A, autoantibody against zinc transporter 8.

### 3.2 Genotype and allele distributions

Genotype information of both T1D subjects (*P* = 0.1176) and control subjects (*P* = 0.8129) did not show departure from the Hardy-Weinberg equilibrium. The genotypic and allelic frequencies of *C1QTNF6* rs229541 as well as the analyses on the dominant model, recessive model and additive model are all shown in **Table 2**. Frequencies of A/A, G/A and G/G genotypes differed between the T1D group and the control group (*P* = 0.0210). In the dominant model, which was adjusted for DR-DQ genotypes, the T1D group exhibited a lower proportion of G allele carriers (*P* = 0.0423, OR 0.82, 95% CI 0.68-0.99). However, this protective effect was not observed for the G/G genotype in the recessive model, which was also adjusted for DR-DQ genotypes (*P* = 0.0587). The minor allele G was less often seen in cases than in controls (26.4% vs. 29.7%, *P* = 0.0084). In the additive model, which calculated the per copy effect of an allele, the protection by minor allele G (*P* = 0.0093) remained statistically significant after adjusting for DR-DQ genotypes (*P* = 0.0167, OR 0.83, 95% CI 0.71-0.97).

**Table 2:** Frequency distributions of *C1QTNF6* rs229541 genotypes and alleles between Chinese patients with T1D and healthy controls.

C1QTNF6 rs229541	Control subjects n = 1282	T1D patients n = 1278	*P*	*P_adj_*	OR_adj_ (95% CI)
Genotypes			0.0210	NA	NA
A/A	635 (49.5)	703 (55.0)			
G/A	532 (41.5)	475 (37.2)			
G/G	115 (9.0)	100 (7.8)			
Alleles			0.0084	NA	NA
A	1802 (70.3)	1881 (73.6)			
G	762 (29.7)	675 (26.4)			
Dominant model			0.0056	0.0423	0.82 (0.68-0.99)
A/A	635 (49.5)	703 (55.0)			
G/A + G/G	647 (50.5)	575 (45.0)			
Recessive model			0.2964	0.0587	0.71 (0.49-1.01)
A/A + G/A	1167 (91.0)	1178 (92.2)			
G/G	115 (9.0)	100 (7.8)			
Additive model			0.0093	0.0167	0.83 (0.71-0.97)
A/A encoded as 0	635 (49.5)	703 (55.0)			
G/A encoded as 1	532 (41.5)	475 (37.2)			
G/G encoded as 2	115 (9.0)	100 (7.8)			

Data are expressed as n (%). Unadjusted genotypic and allelic frequencies were compared using the chi-squared test. In the logistic regression analysis, the G/A and G/G genotypes were encoded as 1, and A/A was encoded as 0 for the dominant model. The G/G genotype was encoded as 1, whereas the A/A and G/A genotypes were encoded as 0 for the recessive model. The G/G genotype was encoded as 2, G/A as 1 and A/A as 0 for the additive model. For the outcome variable, T1D was defined as 1, and control was defined as 0. *P_adj_* and OR_adj:_ To adjust for *DR-DQ* genotypes, they were included in logistic regression as covariates. NA, not available.

### 3.3 Genotype-phenotype correlations

We next analysed the autoantibody status (**Table 3**), age at onset (**Table 4**) and C-peptide levels (**Table 5**) of T1D patients with data broken down by genotypes of *C1QTNF6* rs229541 and adjustment for *DR-DQ* genotypes. No correlation was observed for GADA or IA-2A in any of the 3 genetic models tested (all adjusted *P* values > 0.05). However, in the recessive model, patients with the G/G genotype showed a significantly higher positive rate of ZnT8A than carriers of the A allele. (47.0% vs. 33.5%, *P* = 0.0171, OR 1.88, 95% CI 1.12-3.16). There was no significant association between ZnT8A and *C1QTNF6* rs229541 in the dominant model or in the additive model (both *P* values > 0.05).

**Table 3:** Autoantibody status stratified by *C1QTNF6* rs229541 genotypes in Chinese patients with T1D.

C1QTNF6 rs229541	Negativity of islet autoantibodies	Positivity of islet autoantibodies	*P*	*P_adj_*	OR_adj_ (95% CI)
	GADA (-), n = 125	GADA (+), n = 1151			
Additive model			0.2392	0.2200	0.84 (0.63-1.11)
A/A	66 (52.8)	636 (55.3)			
G/A	44 (35.2)	430 (37.4)			
G/G	15 (12.0)	85 (7.4)			
Dominant model			0.6002	0.5634	0.90 (0.62-1.30)
A/A	66 (52.8)	636 (55.3)			
G/A + G/G	59 (47.2)	515 (44.7)			
Recessive model			0.0712	0.0670	0.57 (0.31-1.04)
A/A + G/A	110 (88.0)	1066 (92.6)			
G/G	15 (12.0)	85 (7.4)			
	IA-2A (-), n = 504	IA-2A (+), n = 494			
Additive model			0.3886	0.3166	1.11 (0.91-1.35)
A/A	275 (54.6)	260 (52.6)			
G/A	192 (38.1)	190 (38.5)			
G/G	37 (7.3)	44 (8.9)			
Dominant model			0.5407	0.5002	1.09 (0.85-1.41)
A/A	275 (54.6)	260 (52.6)			
G/A + G/G	229 (45.4)	234 (47.4)			
Recessive model			0.3659	0.2637	1.31 (0.82-2.09)
A/A + G/A	467 (92.7)	450 (91.1)			
G/G	37 (7.3)	44 (8.9)			
	ZnT8A (-), n = 567	ZnT8A (+), n = 298			
Additive model			0.5967	0.5595	1.07 (0.85-1.34)
A/A	305 (53.9)	166 (55.7)			
G/A	226 (39.9)	101 (33.9)			
G/G	35 (6.2)	31 (10.4)			
Dominant model			0.6101	0.5931	0.93 (0.70-1.23)
A/A	305 (53.9)	166 (55.7)			
G/A + G/G	261 (46.1)	132 (44.3)			
Recessive model			0.0281	0.0171	1.88 (1.12-3.16)
A/A + G/A	531 (93.8)	267 (89.6)			
G/G	35 (6.2)	31 (10.4)			

Data are expressed as n (%) and were computed by logistic regression, in which positivity (encoded as 1) or negativity (encoded as 0) for islet autoantibodies was set as the outcome variable. G/A and G/G genotypes were encoded as 1 and A/A as 0 for the dominant model. The G/G genotype was encoded as 1, whereas the A/A and G/A genotypes were encoded as 0 for the recessive model. The G/G genotype was encoded as 2, G/A as 1 and A/A as 0 for the additive model. *P_adj_* and OR_adj:_ To adjust for *DR-DQ* genotypes, they were included in logistic regression as covariates.

**Table 4:** Age at diagnosis stratified by *C1QTNF6* rs229541 genotypes in Chinese patients with T1D.

C1QTNF6 rs229541	Early disease-onset	Late disease-onset	*P*	*P_adj_*	OR_adj_ (95% CI)
	Age at onset < 15 n = 434	Age at onset ≥ 15 n = 838			
Additive model			0.2268	0.2367	1.12 (0.93-1.36)
A/A	231 (53.2)	467 (55.7)			
G/A	163 (37.6)	311 (37.1)			
G/G	40 (9.2)	60 (7.2)			
Dominant model			0.3953	0.3685	1.12 (0.87-1.44)
A/A	231 (53.2)	467 (55.7)			
G/A + G/G	203 (46.8)	371 (44.3)			
Recessive model			0.1974	0.2540	1.30 (0.83-2.03)
A/A + G/A	394 (90.8)	778 (92.8)			
G/G	40 (9.2)	60 (7.2)			
	Age at onset < 18 n = 543	Age at onset ≥ 18 n = 729			
Additive model			0.2044	0.1921	1.13 (0.94-1.36)
A/A	288 (53.0)	410 (56.2)			
G/A	208 (38.3)	266 (36.5)			
G/G	47 (8.7)	53 (7.3)			
Dominant model			0.2563	0.2160	1.16 (0.92-1.47)
A/A	288 (53.0)	410 (56.2)			
G/A + G/G	255 (47.0)	319 (43.8)			
Recessive model			0.3644	0.4223	1.20 (0.77-1.85)
A/A + G/A	496 (91.3)	676 (92.7)			
G/G	47 (8.7)	53 (7.3)			

Data are expressed as n (%) and were computed by logistic regression, in which early (encoded as 1) or late disease onset (encoded as 0) was set as the outcome variable. G/A and G/G genotypes were encoded as 1 and A/A as 0 for the dominant model. The G/G genotype was encoded as 1, whereas the A/A and G/A genotypes were encoded as 0 for the recessive model. The G/G genotype was encoded as 2, G/A as 1 and A/A as 0 for the additive model. *P_adj_* and OR_adj:_ To adjust for *DR-DQ* genotypes, they were included in logistic regression as covariates.

**Table 5:** Beta-cell function stratified by *C1QTNF6* rs229541 genotypes in Chinese patients with T1D.

C1QTNF6 rs229541	Without beta-cell failure	Beta-cell failure	*P*	*P_adj_*	OR_adj_ (95% CI)
	FCP ≥ 50pmol/L n = 756	FCP < 50pmol/L n = 419			
Additive model			0.0296	0.0296	0.80 (0.65-0.98)
A/A	397 (52.5)	251 (59.9)			
G/A	301 (39.8)	134 (32.0)			
G/G	58 (7.7)	34 (8.1)			
Dominant model			0.0058	0.0058	0.70 (0.54-0.90)
A/A	397 (52.5)	251 (59.9)			
G/A + G/G	359 (47.5)	168 (40.1)			
Recessive model			0.9418	0.9947	1.00 (0.63-1.60)
A/A + G/A	698 (92.3)	385 (91.9)			
G/G	58 (7.7)	34 (8.1)			
	PCP ≥ 100pmol/L n = 686	PCP < 100pmol/L n = 376			
Additive model			0.9362	0.9335	1.01 (0.82-1.25)
A/A	372 (54.2)	214 (56.9)			
G/A	268 (39.1)	123 (32.7)			
G/G	46 (6.7)	39 (10.4)			
Dominant model			0.8591	0.8245	0.87 (0.67-1.14)
A/A	372 (54.2)	214 (56.9)			
G/A + G/G	314 (45.8)	162 (43.1)			
Recessive model					
A/A + G/A	640 (93.3)	337 (89.6)	0.0692	0.0670	1.55 (0.97-2.47)
G/G	46 (6.7)	39 (10.4)			

Data are expressed as n (%) and were computed by logistic regression, in which worse (encoded as 1) or better beta-cell function (encoded as 0) was set as the outcome variable. G/A and G/G genotypes were encoded as 1 and A/A as 0 for the dominant model. The G/G genotype was encoded as 1, whereas the A/A and G/A genotypes were encoded as 0 for the recessive model. The G/G genotype was encoded as 2, G/A as 1 and A/A as 0 for the additive model. *P_adj_* and OR_adj:_ *P* and *OR* were adjusted for HLA information by inclusion of DR-DQ genotypes in logistic regression. To adjust for *DR-DQ* genotypes, they were included in logistic regression as covariates. All analyses in this table were adjusted for disease duration.

When T1D participants were separated into those with childhood onset (age at onset < 15 years) and those without childhood onset (age at onset ≥ 15 years), genotypic information of *C1QTNF6* rs229541 did not alter the risk for early disease onset under all genetic models tested (all *P* values > 0.05), as shown in **Table 4**. Furthermore, when the cut-off point for age at diagnosis was set to 18 years – which is considered the beginning of adulthood among Chinese individuals – there was still no significant association between the *C1QTNF6* polymorphism and susceptibility to early disease manifestation under in dominant model, recessive model or additive model (all *P* values > 0.05).

A fasting C-peptide level < 50 pmol/L or a postprandial C-peptide level < 100 pmol/L was defined as worse beta-cell function, and those with fasting C-peptide ≥ 50 pmol/L or postprandial C-peptide ≥ 100 pmol/L were considered to have better beta-cell function. In the additive model for the analysis of fasting C-peptide, a protective effect was revealed for the G allele (*P* = 0.0296, OR 0.80, 95% CI 0.65-0.98), as shown in **Table 5**. Moreover, in the dominant model, G allele carriers exhibited a dramatically lower frequency of worse beta-cell function than those with the A/A genotype (31.9% vs. 38.7%, *P* = 0.0058, OR 0.70, 95% CI 0.54-0.90). Nonetheless, such a protective effect on beta-cell function was not observed for *C1QTNF6* rs229541 in the analysis of postprandial C-peptide, irrespective of the genetic models assumed (all *P* values > 0.05).

## 4 Discussion

To the best of our knowledge, the first genome-wide association study conducted among Chinese individuals identified BTN3A1, GATA3, and SUOX as susceptibility loci for T1D, in addition to HLA region, *PTPN22*, *CTLA4*, *ERBB3* and *STAT4* (17). The handful of genomic locations confirmed thus far among Chinese patients indicate that the genetic architecture of T1D has yet to be fully elucidated, and several obstacles have hampered the attempt to identify additional determinants of T1D risk. First, given the greatest contribution to T1D by HLA signals, we cannot conclude that certain non-HLA alleles are independently linked to the disease unless we adjust for *DR-DQ* markers. The second problem to be resolved when dealing with genes showing modest effect size is that a sufficient number of samples is necessary to maximize statistical power. Third, considering that T1D can appear at any age and that most new-onset T1D cases in China occur among adults (18), studies that examine only childhood-onset T1D patients can hardly provide a comprehensive interpretation of the genetic background for Chinese individuals. Fourth, as China is a melting pot of various races and because some ethnic minorities in China are genetically closer to Caucasians than to Han individuals (19), the results can be misleading if heterogenous populations are enrolled. The current study enrolled a considerably large sample of genetically homogeneous T1D subjects with Han ethnicity, thus covering all age groups. Additionally, the models examined herein were adjusted for *DR-DQ* genotypes to determine the true effect of *C1QTNF6* rs229541 in Chinese individuals.

As shown in **Table 2**, the genotypes and alleles of rs229541 were heterogeneously distributed between patients and control subjects, with a significant decrease in G allele frequency observed among patients. The additive model has become the most important model for calculating the per copy effect of each allele. In this model adjusted for *DR-DQ* genotypes, the A allele conferred susceptibility to T1D with an OR of 1.20 (OR 0.83 for G allele), in accordance with a previous genome-wide association study (2) in Caucasians (OR 1.15-1.16 for A allele). These findings suggest that *C1QTNF6* rs229541 plays a minor but indispensable role in both ethnic groups. A higher percentage of G allele carriers (dominant model) was observed in controls than in T1D patients, indicating that one copy of minor allele G was sufficient to reduce T1D risk. However, such protection was not confirmed in carriers of the G/G genotype compared to those with the A allele (recessive model), likely due to the risk of the A/A genotype counterbalanced by protection of the G/A genotype when combined and the relatively low counts and frequencies of minor allele G homozygotes in this population.

Full-blown T1D is usually preceded by seroconversion to the presence of autoantibodies against pancreatic islets (1). Both HLA (20) and non-HLA genes (21) exert their function on altering the specificity of the antibody initiating autoimmune process, i.e., GADA or IAA as the first autoantibody. Three islet autoantibodies were routinely detected in our department prior to the diagnosis of classic T1D. Although the data presented here did not support the correlation of *C1QTNF6* rs229541 with GADA or IA-2A, it was intriguing that patients with the susceptible A allele exhibited a lower positive rate of ZnT8A than those homozygous for the G allele. Such a seemingly paradoxical phenomenon could also be seen in the negative association of HLA-A*24 (a well-established HLA Class I allele susceptible to T1D) with IA-2A (22) and ZnT8A (23). E.M. Balke described this inverse relationship as “a delayed antigen spreading of humoral autoimmunity” in carriers of certain risk alleles (24). We next categorized the age at diagnosis into either early or late onset to determine whether the allelic variation in *C1QTNF6* could partially explain the prevalence of late T1D onset in Chinese patients. Regardless of whether the cut-off age was 15 years or 18 years and regardless of the genetic models used, genotypic frequencies of rs229541 did not significantly differ between the two groups with distinct onset ages. Both FCP and PCP were measured in this research to evaluate the beta-cell function of patients with T1D, and a lower frequency of FCP < 50 pmol/L was observed for patients with the protective G allele in comparison with those carrying the A/A genotype. Accumulating evidence gathered from multiple populations supports the effect of polymorphisms in non-HLA genes, such as *PTPN22*, *FCRL3* (25) and vitamin D receptor gene (26), on modulating beta-cell function in T1D subjects. Nonetheless, one major limitation of the current research was that only cross-sectional data of islet autoantibodies and C-peptide were available here. Therefore, we were not able to investigate whether the *C1QTNF6* polymorphism alone or in combination with other genetic variants could predict the appearance of autoantibodies, the progression to clinical T1D or the fast decline in beta-cell function in Chinese patients.

Fortunately, studies of Caucasian populations managed to build prospective cohorts that carefully followed up genetically susceptible children from birth to the initiation of islet autoimmunity and ultimately to T1D diagnosis; both encouraging and negative results have been reported. For instance, *C1QTNF6* rs229541 has been shown to predict the risk of islet autoimmunity instead of progression to T1D in DAISY children (27), whereas the methylation of this gene was not correlated with T1D development in another study conducted by the same group (28). The MIDIA study (29) detected enteroviral RNA in stool samples from Norwegian children, genotyped both T1D susceptibility SNPs and those within innate immune genes and found that the protective genotype of *C1QTNF6* rs229541 was related to a lower enterovirus frequency, thus emphasizing the interplay between genetics and environmental factors when scrutinizing the etiology of T1D.

As C1QTNF6 plays an active role in a wide variety of endocrine and inflammatory pathways, C1QTNF6-related pathophysiological processes or diseases include but are by no means limited to T1D. Polycystic ovary syndrome (PCOS) is a common reproductive endocrine disorder, the pathogenesis of which features low-grade chronic inflammation. S. Yan and his colleagues found that the expression of C1QTNF6 was elevated in serum from both PCOS patients and PCOS mice compared to the corresponding controls. They further demonstrated that the levels of proinflammatory factors, including CRP, IL6 and TNFα, were increased in C1QTNF6-overexpressing PCOS mice and that this protein regulated the inflammatory response through the AKT/NF-κB signaling pathway (30). J. Wang revealed that miR-29b-3p modulated particulate matter-induced inflammatory responses via the C1QTNF6/AMPK pathway (31), which provided new insights into the molecular mechanisms behind chronic inflammation in respiratory diseases. A study by the Institute of Medical Science from the University of Tokyo showed a higher expression of C1QTNF6 in mouse models with rheumatoid arthritis. C1QTNF6 (-/-) mice were extremely prone to induced arthritis because of augmented complement activation. C1QTNF6 specifically inhibited the alternative pathway in the complement system by competing against factor B for C3 (H2O) binding. Intra-articular injection of arthritis-induced mice with recombinant human C1QTNF6 cured the disease (32), which suggested that this protein is a novel target for the treatment of inflammatory diseases. Researchers from Israel proposed C1QTNF6 as a link between immunity and metabolism, as this protein responds to a rapid change in the calorie intake of mice, triggering a “physiological inflammatory response” that suppresses the expansion of adipose tissue (33). For the development of malignancies, upregulation of C1QTNF6 was observed for stage I lung adenocarcinoma in contrast to the adjacent noncancer tissues. Knockdown of C1QTNF6 markedly dampened cell proliferation, migration and invasion, which was negatively regulated by miR-29a-3p (34). A similar role of C1QTNF6 was also recognized in oral squamous cell carcinoma, as C1QTNF6 knockdown led to cell cycle arrest at phase G2/M and enhancement of cancer cell apoptosis (35). Overall, the publications described above indicate that C1QTNF6 is actively engaged in a broad spectrum of pathophysiological events, including immunity directed towards either self-tissues or external stimuli, lipid metabolism and homeostasis as well as oncogenesis across multiple disciplines.

Additionally, the studies mentioned further suggest that the polymorphism rs229541 in C1QTNF6 is associated with immune responses in T1D. C1QTNF6’s involvement in the complement system may also affect the clearance of apoptotic cells and the resolution of inflammation. By regulating complement activation, C1QTNF6 could prevent excessive tissue damage and autoimmunity, potentially limiting the progression of β-cell destruction in T1D patients. The G allele of rs229541 may alter immune responses by affecting the function of antigen-presenting cells or regulatory T cells, both of which are essential for maintaining immune tolerance (24, 30). Our study found that the G allele of rs229541 in C1QTNF6 is positively correlated with ZnT8A positivity, which may be related to the “delayed antigen spreading of humoral autoimmunity” mentioned earlier. C1QTNF6 may impact the development of islet autoantibodies by regulating B cell activation and plasma cell differentiation (36). The immune-modulatory role of C1QTNF6 has been recognized in other autoimmune diseases, such as rheumatoid arthritis, where its expression and function in immune cells contribute to either promoting or suppressing autoimmunity, depending on the genetic context (32). Moreover, C1QTNF6 may regulate the inflammatory process through key immune signaling pathways such as NF-κB and AKT, playing a crucial role in the modulation of cytokine levels and immune cell activation (37). By influencing these pathways, C1QTNF6 could help shape the immune microenvironment within the pancreatic islets, potentially altering the initiation and progression of autoimmune responses. These findings suggest that C1QTNF6 could have a broader impact on the regulation of immune responses in T1D, making it an important candidate for further investigation in the context of genetic susceptibility and immune tolerance.

Taken together, the data presented herein reveal the role of the *C1QTNF6* polymorphism rs229541 in the pathogenesis of T1D of Chinese origin, and it also modulates biochemical profiles, including islet autoantibodies and C-peptide levels, independent of DR-DQ genotypes. However, it is important to interpret these results with caution, as several limitations should be considered. First, the sample population was limited to Han Chinese individuals, which may affect the generalizability of the results to other ethnic groups. Further studies involving diverse populations and larger sample sizes are needed to confirm these findings. Second, environmental factors such as viral infections, diet, and other exposures were not accounted for, despite their known influence on T1D development. Future studies should incorporate these factors to better understand the genetic-environmental interactions in T1D. Lastly, as mentioned earlier, the cross-sectional design limits our ability to infer causality. Longitudinal studies are necessary to explore the temporal relationship between genetic variations and disease onset. Future research should examine whether this gene polymorphism aids in predicting the clinical course of T1D from genetic susceptibility and seroconversion to clinical manifestation in non-Caucasian populations.

## Data Availability

The original contributions presented in the study are included in the article/**Supplementary Material**. Further inquiries can be directed to the corresponding authors.

## Supplementary material

Supplementary Figure 1 Distribution of C-peptide levels in Chinese T1D patients stratified by C1QTNF6 rs229541 genotypes. This boxplot presents the distribution of C-peptide levels (pmol/L) among Chinese T1D patients, comparing individuals carrying the G/A and G/G genotypes (combined) with those harboring the A/A genotype. Group differences were evaluated using the Mann-Whitney U test. Statistical significance is denoted as *P < 0.05, **P < 0.01, and ***P < 0.001.
