# Supplementary figures and images for: Association of C1QTNF6 gene polymorphism with risk and clinical features of type 1 diabetes in Chinese: implications for ZnT8A and beta-cell function
Source: Front Immunol. 2025 Apr 9;16:1551552. doi: 10.3389/fimmu.2025.1551552 (PMC12014654; doi:10.3389/fimmu.2025.1551552)

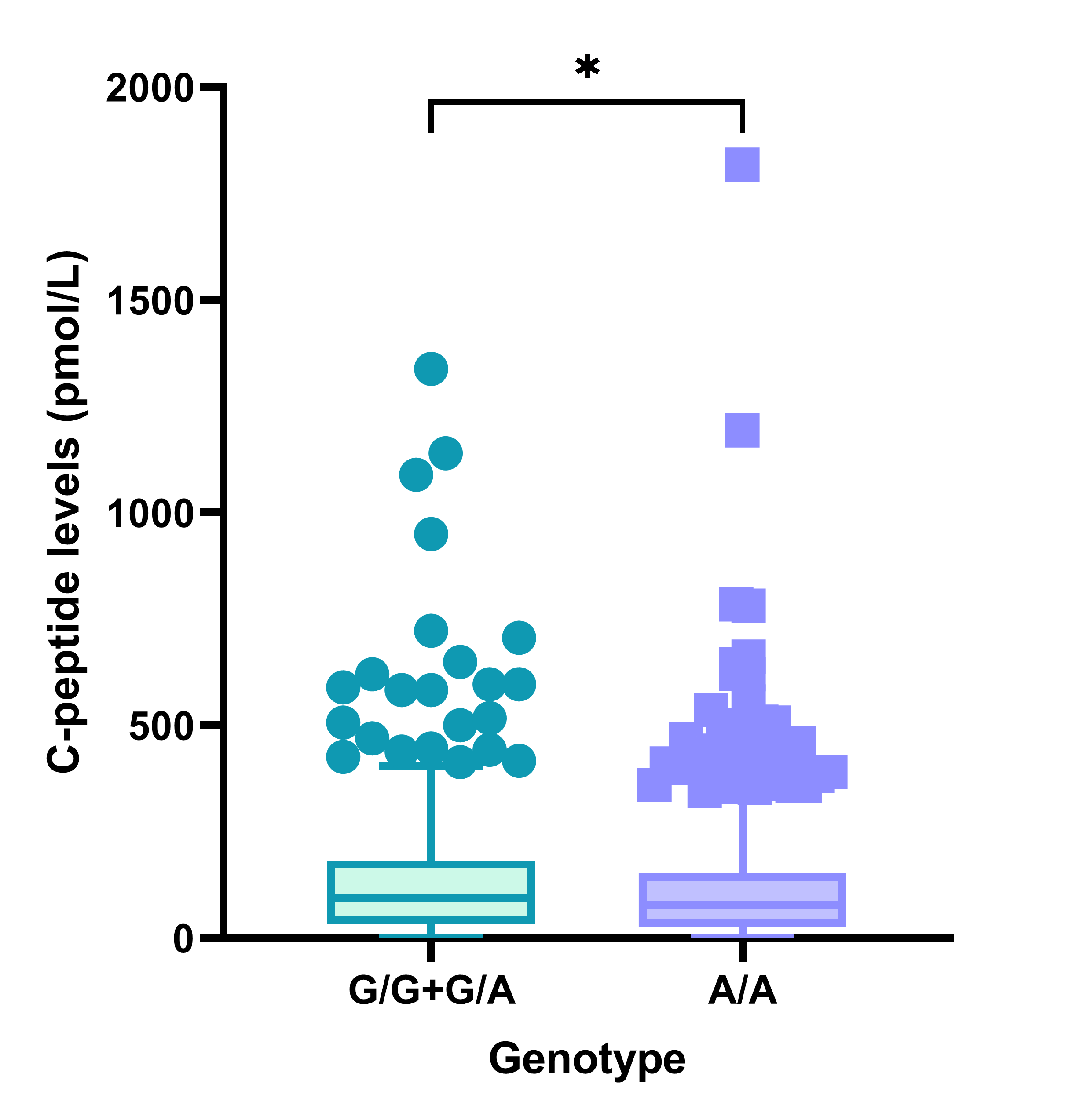

Supplement: Supplementary Figure 1 — Distribution of C-peptide levels in Chinese T1D patients stratified by C1QTNF6 rs229541 genotypes. This boxplot presents the distribution of C-peptide levels (pmol/L) among Chinese T1D patients, comparing individuals carrying the G/A and G/G genotypes (combined) with those harboring the A/A genotype. Group differences were evaluated using the Mann-Whitney U test. Statistical significance is denoted as *P < 0.05, **P < 0.01, and ***P < 0.001. [file Image1.tif]
